# Rootstock genotype shapes vine functional traits and associated microbiomes, with predominant shifts in the phyllosphere

**DOI:** 10.1007/s00425-026-05132-6

**Published:** 2026-09-08

**Authors:** Ginevra Canavera, Filippo Vaccari, Matteo Gatti, Edoardo Puglisi, Tommaso Frioni

**Affiliations:** 1 Department for Sustainable Food Process, Università Cattolica del Sacro Cuore, Via Emilia Parmense 84, 29122 Piacenza, Italy; 2 Department of Sustainable Crop Production, Università Cattolica del Sacro Cuore, Via Emilia Parmense 84, 29122 Piacenza, Italy; 3 National Research Centre for Agricultural Technologies (Agritech Foundation, PNRR M4C2), Naples, Italy

**Keywords:** Bacterial diversity, Drought tolerance, Fungal diversity, Host genotype, Plant–microbe interaction, Vineyard ecology

## Abstract

**Main conclusion:**

Rootstock genotype shaped grapevine physiology and associated microbial communities, with the strongest compositional shifts consistently observed in the phyllosphere across contrasting growing seasons.

**Abstract:**

Rootstocks contribute to grapevine adaptation to biotic and abiotic stresses, including drought, and influence associated microbial communities relevant to nutrient dynamics and stress resilience. However, integrated analyses of rhizosphere and phyllosphere microbiomes remain limited, particularly for newly developed drought-tolerant rootstocks (M2 and M4). This study tested the hypothesis that rootstock genotype influences vine physiology and vegetative growth while shaping microbial community composition and functional potential in the rhizosphere and phyllosphere. The study was conducted from 2023 to 2024 on *Vitis vinifera* cv. Barbera grafted onto six rootstocks (1103 Paulsen, 140 Ruggeri, SO4, Kober 5BB, M2, and M4). Phyllosphere and rhizosphere samples were collected at grapevine flowering and veraison, together with measurements of leaf gas exchange and stem water potential (Ψ_stem_). Vegetative growth was assessed at the end of each season through leaf area, node number, and pruning weight. Microbial communities were profiled through 16S rRNA gene and ITS amplicon sequencing. Across both years, despite contrasting weather conditions, rootstock genotype affected vine performance, while the strongest differences in microbial communities were observed in the phyllosphere. Notably, M2 and M4 maintained higher Ψ_stem_ than the other rootstocks, indicating greater drought tolerance. Phyllosphere microbiome composition appeared to be shaped by rootstock-related differences in vine physiology and vegetative growth, likely through local microenvironmental changes affecting microbial assembly. Expanding integrative research linking plant physiology and microbiome ecology could clarify how rootstock-mediated traits influence grapevine-associated microbiomes and their potential contribution to microbial terroir, ultimately supporting more resilient and sustainable viticulture under climate change.

**Supplementary Information:**

The online version contains supplementary material available at https://doi.org/10.1007/s00425-026-05132-6.

## Introduction

*Vitis vinifera* L. is widely grafted onto rootstocks derived from other *Vitis* species, which confer resistance to phylloxera and can enhance tolerance to abiotic stresses, nematodes, and nutrient-poor soils, ultimately influencing scion vigor, yield, and grape quality (Warschefsky et al. 2016). Traditional grapevine rootstocks primarily originate from hybrids of three American species, *Vitis riparia*, *Vitis rupestris*, and *Vitis berlandieri*, valued for their resistance traits and strong intra- and interspecific hybridization compatibility. However, the limited number of available genotypes and the need for rootstocks better adapted to European growing conditions have encouraged the development of new hybrids, including crosses with *Vitis vinifera* (Bianchi et al. 2018).

In 2014, a new generation of rootstocks (the “M” series: M1, M2, M3, and M4), developed by the Department of Agricultural and Environmental Sciences (DISAA) at the University of Milan (Italy), was officially registered in the National Register of Vine Varieties (RNVV). Among them, M2 and M4 show moderate tolerance to salinity, while M4 also exhibits high drought tolerance (Porro et al. 2013; Meggio et al. 2014). This trait is particularly relevant in rainfed vineyards, where the increasing frequency of water-scarcity events poses a major challenge for Mediterranean viticulture, leading to leaf photoinhibition, yield losses, and declines in fruit quality (Poni et al. 2018). These rootstocks have been evaluated for their physiological and agronomic performance under controlled drought and salinity stress, confirming their potential ability to enhance the scion’s performance under water deficit compared with traditional rootstocks (Meggio et al. 2014; Bianchi et al. 2020; Frioni et al. 2020; Rius-Garcia et al. 2025). However, their responses with the scion cv. Barbera have not yet been studied, and their performance under field conditions remains poorly documented.

Plants are increasingly viewed through an integrated plant-microbiome perspective, in which associated microbial communities can contribute to host performance and adaptation. Accordingly, rootstocks may shape grapevine traits not only by modulating growth and physiology, but also by influencing the composition and functional potential of the grapevine-associated microbiome (Lailheugue et al. 2024). This relationship is particularly relevant because shifts in microbial community structure can modulate plant physiology and metabolism across both below- and above-ground organs, with potential consequences for grape and wine quality (Trivedi et al. 2020; Borghi et al. 2024). In turn, the grapevine microbiome both reflects plant-environment interactions and contributes to key ecological functions, including nutrient cycling, stress alleviation, and disease suppression, ultimately supporting vine health and resilience. However, studies investigating rootstock-associated microbiome variation have mainly focused on below-ground compartments, such as the rhizosphere, root endosphere and bulk soil (D’Amico et al. 2018; Marasco et al. 2018; Berlanas et al. 2019; Zuzolo et al. 2023), whereas the above-ground, such as phyllosphere, remains comparatively poorly explored (Zhang et al. 2025).

Recent advances in high-throughput sequencing technologies have greatly expanded our ability to characterize plant-associated microbial communities using culture-independent approaches, which characterize microbial communities directly from environmental samples without prior cultivation, providing insights into their taxonomic composition and potential functional roles (Nam et al. 2023; Rai et al. 2023). These approaches have revealed that the grapevine microbiome is highly compartmentalized, with distinct microbial assemblages inhabiting different plant organs depending on their structure, microenvironment, and resource availability (Martins et al. 2013; Swift et al. 2021). The rhizosphere and phyllosphere represent two major microbial habitats that differ in environmental exposure and nutrient availability, yet both contribute to vine performance and stress resilience by enhancing nutrient acquisition, mitigating abiotic stress, and suppressing pathogens (Gilbert et al. 2014; Hardoim et al. 2015).

Rootstock genotype can influence these plant-associated microbial communities through its effects on root architecture, physiology, and plant metabolism. Because rootstocks and scions have distinct genetic backgrounds, their associated microbiomes may differ substantially between below- and above-ground compartments (Marasco et al. 2018; Dries et al. 2021; Lailheugue et al. 2024). However, grapevine-associated microbiomes are also strongly shaped by environmental factors, including year and phenological stage (Redford and Fierer 2009; Liu and Howell 2020). Therefore, rootstock effects were evaluated within comparable experimental context. Moreover, the newly developed M2 and M4 rootstocks remain largely unexplored with respect to their associated microbiomes, highlighting the need for further investigation. In the present study, we hypothesized that: (i) rootstock genotype influences vine physiological performance and vegetative growth; and (ii) these rootstock-associated differences are accompanied by shifts in the composition and functional potential of microbial communities in both the rhizosphere and phyllosphere.

## Materials and methods

### Experimental design and sample collection

The study was conducted in 2023 and 2024 in a north–south-oriented vineyard planted in 2017 at the “I Salici” estate in Donceto, Italy (44°49' N, 9°29' E), within the Colli Piacentini wine district. *Vitis vinifera* L. cv. Barbera vines were grafted onto three *Vitis berlandieri* × *Vitis riparia* rootstocks, Kober 5BB (K5BB), 420 A, and Selezione Oppenheim n° 4 (SO4), one *Vitis berlandieri* × *Vitis rupestris* rootstock, 1103 Paulsen (1103P), and two recently bred complex *Vitis* hybrids, M2 [(*V. berlandieri* × *V. riparia*) × (*V. vinifera* × *V. berlandieri*) and M4 [(*V. vinifera* × *V. berlandieri*) × *V. berlandieri*]. These six scion-rootstock combinations (Barbera grafted to K5BB, 420 A, SO4, 1103P, M2, and M4) represented the treatments in this study. Vines were planted at a spacing of 2.5 × 1.60 m (between rows × within-row distance) and trained to a unilateral Guyot with approximately 10 nodes on the primary cane. Standard organic viticulture practices, in accordance with regional regulations, were implemented throughout the study. In each year, copper and sulfur were applied seven times between May and early July. Control of the Flavescence dorée vector was carried out using a treatment containing sweet orange essential oil. Each treatment consisted of one row of 30 vines (180 vines in total). Three blocks were defined within each row, and three vines per block were randomly selected, yielding nine vines per treatment for sampling and physiological measurements.

Phenological stages were assigned based on field observations of grapevine development according to the modified BBCH scale (Lorenz et al. 1995): budburst (BBCH09), flowering (BBCH68), and veraison (BBCH81).

Meteorological data, including minimum, mean, and maximum air temperature (°C), daily rainfall (mm), minimum, mean, and maximum relative humidity (%), and solar irradiation (MJ m^−2^), were recorded throughout each growing season using a Sencrop weather station located within the vineyard. Growing degree days (GDD) and accumulated rainfall were calculated from budburst to veraison (second sampling date) in both years. For comparison between phenological stages, the same variables were also calculated separately for the periods between budburst and flowering (first sampling date) and between flowering and veraison. Vapor pressure deficit (VPD) was calculated as:

$$VPD={e}_{s}\left(\mathrm{T}\right)*\left(1-\frac{RH}{100}\right)$$

where ${e}_{s}$ (T) is the saturation vapor pressure (kPa) estimated from air temperature (T, °C) using the Tetens equation:

$${e}_{s}\left(\mathrm{T}\right)=0.61078*exp\frac{17.27*\mathrm{T}}{\mathrm{T}+237.3}$$

with RH representing relative humidity (%). Together with irradiance (I), the average values for the 10 days preceding each sampling date were determined. Soil and leaf samples were collected at two key phenological stages: flowering (DOY 152 in 2023 and DOY 157 in 2024) and veraison (DOY 219 in 2023 and DOY 225 in 2024).

Material from the three vines within the same block was pooled to form a composite sample, representing one biological replicate. This resulted in three biological replicates per treatment, per sampling date, and per source (soil and leaf). In total, 36 samples were collected at each sampling date (18 soil and 18 leaf samples), corresponding to 72 samples per year (36 soil and 36 leaf samples).

Soil sampling involved extracting cores at a depth of 25–30 cm, approximately 20 cm from the vine trunk, with two subsamples collected per vine. Samples were immediately placed in sterile bags on dry ice during field collection and subsequently stored at − 20 °C. In the laboratory, roots recovered from soil cores were gently shaken to eliminate bulk soil. The tightly adhering soil particles were then detached from the root surface using sterile forceps to isolate the rhizosphere fraction, following the protocol outlined by Ambrosini and Passaglia (2017).

Leaves were collected by sampling five healthy leaves per vine from nodes 4 to 7, as mid-canopy leaves are fully expanded and physiologically stable, ensuring consistent sampling, using the same storage procedure as for soil samples. The epiphytic phyllosphere fraction was extracted following the method described by Perazzolli et al. (2014). Briefly, leaves from each biological replicate were washed in 300 mL of an isotonic solution containing 0.01% Tween 80 for 15 min using a BagMixer 400S (Interscience, Saint Nom la Bretêche, France). The wash solution was then centrifuged at 3580 x g for 20 min and the resulting pellet was used for DNA extraction by resuspending it in the buffer provided with the extraction kit.

### Grapevine physiological measurements and vegetative growth

Single-leaf gas exchange was measured only during the second rhizosphere and phyllosphere sampling, whereas Ψ_stem_ was measured at both sampling dates. The gas exchanges readings were recorded around midday (11:30–13:30 solar time) on clear days under saturating light conditions (PAR > 1200 µmol m^−2^ s^−1^) by using a portable, open-system, LCA-3 infrared gas analyzer (ADC BioScientific Ltd., Herts, UK), equipped with a broad leaf chamber featuring a 6.25 cm^2^ window, on one mature well-exposed primary leaf (nodes 4–7) per vine (totally 9 per treatment). The assimilation rate (A), transpiration rate (E), and stomatal conductance (g_s_) were calculated from inlet and outlet CO_2_ and H_2_O relative concentrations. Extrinsic water use efficiency (WUE_e_) was then determined as A/E. Subsequently, the same leaves were covered by aluminum, and after 45 min, stem water potential (Ψ_stem_) was measured using a Scholander pressure chamber (3500 Model, Soilmoisture Equip. Corp., Santa Barbara, CA, USA).

Total leaf area (LA) per vine was estimated according to Gatti et al. (2017). Briefly, at harvest, one representative shoot per tagged vine was collected, and all main and lateral leaves were counted and measured using a leaf-area meter (LI-3000A, LI-COR Biosciences, Lincoln, NE, USA). The total number of nodes per vine was recorded after leaf fall, and LA was calculated by combining mean leaf area with node number, distinguishing between main and lateral shoots. One-year-old pruning weight was taken during winter pruning as another indicator of vegetative growth.

### Microbial analyses

#### DNA extraction and 16S and ITS amplicon sequencing

DNA was extracted from homogenized 0.25 g of rhizosphere and phyllosphere material using the FastDNA SPIN Kit for Soil (MP Biomedicals, Irvine, CA, USA) according to the manufacturer’s protocol. DNA yields were quantified with the Quant-iT™ High Sensitivity dsDNA Assay Kit (Invitrogen, Paisley, UK) and measured using a Qubit 2.0 fluorometer (Invitrogen).

Bacterial community analysis was performed by amplifying the V3-V4 hypervariable region of the 16S rRNA gene with primers 343 F (5′-TACGGRAGGCAGCAG-3′) and 802R (5′-TACNVGGGTWTCTAATCC-3′), following the PCR mixture preparation, cycling conditions, and nested PCR protocol described by Vaccari et al. (2023). In brief, each PCR reaction (25 μL total volume) contained 12.5 μL Phusion Flash Master Mix, 1.25 μL of each primer (10 μM), 2 ng DNA template, and nuclease-free water. The amplification program included 95 °C for 5 min; 20 cycles of 95 °C for 30 s, 50 °C for 30 s, and 72 °C for 30 s, followed by a final extension at 72 °C for 10 min. For multiplexing, a nested PCR was performed using the products of the first reaction as templates. A negative control for PCR amplification was included using nuclease-free water instead of template DNA.

Fungal community analysis was performed through amplification of the ITS1 and ITS2 regions of the ribosomal RNA gene using the universal primers ITS1 (5′-TCCGTAGGTGAACCTGCGC-3′) and ITS2 (5′-GCTGCGTTCTTCATCGATGC-3′), with an analogous nested PCR workflow. The amplification program consisted of 94 °C for 4 min, followed by 28 cycles of 94 °C for 30 s, 56 °C for 30 s, and 72 °C for 1 min, and a final extension at 72 °C for 7 min.

PCR products were purified via solid-phase reversible immobilization (SPRI) using the Agencourt AMPure XP kit (REF A63880, Beckman Coulter). Amplicon libraries were prepared using the TruSeq DNA Sample Preparation Kit (Illumina) and sequenced by Novogene UK (Cambridge, UK) on an Illumina NovaSeq 6000 platform, generating 250 bp paired-end reads. Sequence data were quality-checked, denoised, and filtered using the DADA2 plugin in QIIME2 (https://docs.qiime2.org/2024.10/plugins/available/dada2/). Taxonomic assignment was performed using the SILVA (v138.2) reference database for bacteria and the UNITE (v9.0) database for fungi. To ensure consistency with the amplified regions, full-length 16S sequences from SILVA were trimmed to the primer-defined V3–V4 region, and full-length ITS sequences from UNITE were trimmed to the primer-defined ITS1-ITS2 region using Cutadapt (https://docs.qiime2.org/2024.10/plugins/available/cutadapt/).

#### PICRUSt2 and FUNGuild predictive analyses

As amplicon sequencing does not directly measure microbial functions or ecological roles, the following analyses provide predictive estimates rather than direct measurements. Bacterial functional profiles were inferred using PICRUSt2 (https://github.com/picrust/picrust2; v2.5.1), which predicts the abundance of enzymes and metabolic pathways based on 16S rRNA gene data. Pathway annotation was performed using the Kyoto Encyclopedia of Genes and Genomes (KEGG; https://www.genome.jp/kegg/) and MetaCyc. Fungal trophic modes and guilds were inferred at the genus level using FUNGuild database (Nguyen et al. 2016). Only “probable” and “highly probable” assignments were retained; ASVs with multiple classifications were designated as “multi-affiliated”, while unassigned ASVs were excluded.

### Statistical analyses

Grapevine physiological and vegetative growth data were subjected to a one-way analysis of variance (ANOVA). When the F-test was significant (*p* < 0.05), means separation among treatments was performed using the Student–Newman–Keuls (SNK). Microbial abundance and diversity analyses were performed using MicrobiomeAnalyst (Lu et al. 2023) and Rstudio software (v4.5.1). To obtain an overall assessment of the main drivers structuring microbial communities, alpha diversity (Shannon index; Welch’s ANOVA) and beta-diversity (Bray–Curtis dissimilarity followed by PERMANOVA using the adonis2 function in the vegan package) were analyzed using a global model including treatment, compartment, and phenological stage (marginal tests). Given the strong effects of year, phenological stage, and plant compartment on microbial community composition, the dataset was stratified by these factors, and rootstock effects were assessed within each subset separately. The ASV table was rarefied to the minimum sequencing depth and subsequently normalized using total sum scaling (TSS).

Relative abundance profiles and stacked bar plots at the genus level were generated using the MicrobiomeAnalyst platform. The 10 most abundant taxa per dataset were displayed based on mean relative abundance, while the remaining taxa were grouped as “Others”. α-diversity indices (Chao1 richness estimator, Shannon diversity, and Simpson index) were calculated at the ASV level using the microeco R package. Differences among treatments were evaluated using Welch T-test/ANOVA and post hoc pairwise test (α = 0.05). Beta diversity was assessed in MicrobiomeAnalyst using Bray–Curtis dissimilarity, visualized by principal coordinates analysis (PCoA), and statistically evaluated through PERMANOVA within each experimental subset. Biomarker identification was performed using the linear discriminant Analysis Effect Size (LEfSE) algorithm implemented in the microeco package, applying a Kruskal–Wallis test (*p* < 0.05) followed by linear discriminant analysis (LDA) to estimate effect sizes. Relationships between microbial community structure and grapevine physiological variables were assessed using the envfit and Mantel tests implemented in the vegan package (v2.6-4), based on Bray–Curtis dissimilarities. Each physiological variable was tested individually to evaluate its marginal contribution. *p* values were adjusted for multiple testing using the false discovery rate (FDR) method. For PICRUSt analysis, statistical comparisons were conducted in STAMP (https://beikolab.cs.dal.ca/software/STAMP; v2.3.1) using one-way ANOVA followed by Tukey–Kramer post hoc tests, with Benjamini–Hochberg FDR correction applied to control for multiple testing. When appropriate, pairwise comparisons among rootstocks were performed using Welch’s t-test. Functional pathways were reported according to the MetaCyc classification.

## Results

### Seasonal climatic variables

Table 1 reports the climatic variables recorded at the vineyard site in 2023 and 2024. Growing degree days (GDD), which reflects heat accumulation, differed between seasons, with values about 15% higher from budburst to flowering in 2024 compared to 2023. Rainfall also showed marked seasonal variation, with cumulative precipitation from budburst to flowering nearly doubled in 2024 (314 mm) than 2023 (156 mm), and by veraison reaching 476 mm in 2024, more than twice the amount recorded in 2023 (229 mm). These contrasting precipitation patterns, together with higher mean temperatures and lower relative humidity in 2023, resulted in a higher average vapor pressure deficit (VPD) at veraison (1.30 kPa) compared with 2024 (0.92 kPa).

**Table 1:** Climatic variables recorded in 2023 and 2024 for the periods from budburst to flowering (BB-FL) and from flowering to veraison (FL-VE) at the vineyard site

Year	Rainfall^a^		GDD^a^		Irradiance^a^		VPD^a^	
	BB-FL^b^	FL-VE^b^	BB-FL	FL-VE	a10d-FL^c^	a10d-VE^c^	a10d-FL	a10d-VE
2023	156.33	72.79	282.67	902.55	23.31	21.33	0.63	1.30
2024	314.79	161.69	325.57	936.17	19.43	21.08	0.50	0.92
2024	314.79	161.69	325.57	936.17	19.43	21.08	0.50	0.92

^a^Variables include rainfall (mm), growing degree days (GDD), irradiance (MJ/m^2^), and vapor pressure deficit (VPD, kPa)

^b^BB, FL and VE refer to BudBurst, FLowering and VEraison, respectively

^c^a10d-FL and a10d-VE indicate the average values recorded in the 10 days preceding the flowering and veraison sampling dates, respectively

### Leaf gas exchange, plant water status and vegetative parameters

Physiological parameters and vegetative growth traits differed significantly among treatments and between years (Tables 2 and 3, respectively). At flowering, Ψ_stem_ did not differ among rootstocks (data not shown), with an average across treatments of − 0.6 MPa. By veraison, however, rootstock-specific differences in grapevine water status became evident (Table 2). In 2023, most physiological parameters did not vary significantly among rootstocks, except for g_s_, which was highest in M4 (47.5 mmol m^−2^ s^−1^), and for Ψ_stem_, which differed significantly among treatments (*p* < 0.001). M4 and M2 maintained less negative Ψ_stem_ values (− 1.0 and − 1.2 MPa, respectively), while SO4, 1103P, and 420 A reached − 1.4 MPa. In the same year, M2 and M4 also showed higher leaf area (LA) compared with K5BB (Table 3) and a greater number of nodes per vine (260 and 259, respectively) than 420 A and K5BB (190 and 156, respectively; *p* < 0.001, Table 3). No significant differences were observed in pruning weight.

**Table 2:** Plant water status and leaf gas exchanges parameters taken at DOY 219 and 225 in 2023 and 2024, respectively across the treatments

Year/treatment	2023					2024				
	A^a^	E^a^	gs^a^	WUEe^a^	Ψ_stem_^a^	A	E	gs	WUEe	Ψ_stem_
M2	3.8	2.0	42ab	1.9	−1.2a	10.4ab	3.3b	160b	3.2a	−1.0a
M4	4.4	2.1	47a	2.1	−1.1b	12.2a	5.6a	280a	2.2b	−0.9a
SO4	3.2	1.6	34b	2.1	−1.4c	6.7d	3.3b	93c	2.1b	−1.1b
1103P	3.3	1.5	32b	2.2	−1.4c	10.6ab	5.4a	175b	2.0b	−0.9a
420°	3.4	2.1	41ab	1.7	−1.4c	8.8bc	5.3a	171b	1.6b	−1.1b
K5BB	3.5	2.1	41ab	1.7	−1.4c	7.3bcd	4.5a	128bc	1.6b	−1.1b
Significance	*ns*	*ns*	*	*ns*	***	***	***	***	***	***

Within each column, different letters indicate statistically significant differences according to the SNK test (*p* < 0.05; *n* = 9). * *p* < 0.05, *** *p* < 0.001; *ns* not significant

^a^Mean leaf assimilation (A, µmol m^−2^ s^−1^), mean leaf transpiration rate (E, mmol m^−2^ s^−1^), mean leaf stomatal conductance (g_s_, mmol m^−2^ s^−1^), extrinsic water use efficiency (WUEe, A/E) and midday leaf stem water potential (Ψ_stem_, MPa)

**Table 3:** Vegetative parameters recorded at the end of the 2023 and 2024 growing seasons

Year/treatment	2023			2024		
	Nodes^a^	LA^a^	PW^a^	Nodes	LA	PW
M2	260a	3.18a	0.62	469a	5.33a	1.16a
M4	259a	2.96a	0.63	329b	4.1ab	0.97ab
SO4	206ab	2.53ab	0.39	261b	3.11b	0.80ab
1103P	210ab	2.60ab	0.81	363b	4.12ab	1.03ab
420A	190b	2.45ab	0.55	369b	4.48ab	0.94ab
K5BB	156b	2.08b	0.50	159c	1.99c	0.43b
Significance	***	*	*ns*	***	***	*

Within each column, different letters indicate statistically significant differences according to the SNK test (*p* < 0.05; *n* = 9). * *p* < 0.05, *** *p* < 0.001; *ns* not significant

^a^Total nodes per vine (Nodes), leaf area (LA, m^2^), and pruning weight (PW, kg vine⁻^1^)

In 2024, when vines were overall under light water deficit, significant differences were detected across all parameters. M4 displayed the highest photosynthetic rate (12.2 µmol CO₂ m^−2^ s^−1^), transpiration (5.61 mmol H_2_O m^−2^ s^−1^), and stomatal conductance (280 mmol m^−2^ s^−1^), with water use efficiency (WUEe) comparable to most rootstocks, except for M2, which exhibited higher WUEe (3.19) than the other rootstocks. In contrast, SO4 showed the lowest gas-exchange rates (*A* = 6.7 µmol CO₂ m^−2^ s^−1^ and *g*_s_ = 93 mmol m^−2^ s^−1^). Ψ_stem_ showed significant differences, ranging from − 0.9 MPa in M2, 1103P, and M4 to − 1.1 MPa in 420 A, K5BB, and SO4. In this year, differences in LA were more pronounced (*p* < 0.001), with M2 showing higher values than SO4, while K5BB remained the lowest across treatments. Similarly, M2 exhibited a higher number of nodes per vine (469 vs. 159) and greater pruning weight (1.16 vs. 0.43 kg vine⁻^1^) compared with K5BB (*p* < 0.05, Table 3).

### Global effects of rootstock, plant compartment, and phenological stage on grapevine-associated bacterial and fungal communities

After quality filtering and removal of chimeric, mitochondrial, and chloroplast sequences, 2,973,133 and 1,984,463 bacterial 16S rRNA reads were retained for 2023 and 2024, respectively, while 1,795,419 and 2,121,324 fungal ITS sequences were obtained for the same years. In both years, bacterial Shannon diversity differed significantly between compartments, with consistently higher values in the rhizosphere (mean 5.28 and 5.02 in 2023 and 2024, respectively) compared to the phyllosphere (mean 3.70 and 2.32, respectively). In 2023, phyllosphere diversity increased significantly from flowering to veraison, whereas no phenological effect was detected in 2024.

Fungal diversity showed a similar compartment-dependent pattern, with higher Shannon values in the rhizosphere (mean 2.87 and 2.24 in 2023 and 2024, respectively) than in the phyllosphere (mean 2.32 and 1.39, respectively). A significant increase from flowering to veraison was observed in the phyllosphere in 2023 only. PERMANOVA (Adonis2) confirmed compartment as the dominant driver of community structure in both years (data not shown). In 2023, it explained 50% and 27% of the variation in bacterial and fungal communities, respectively (*p* = 0.001), while the phenological stage exerted a secondary but significant effect. Treatment was not significant in the full model but emerged in compartment-specific analyses.

In 2024, the compartment effect was even stronger, explaining 77.7% and 68.6% of bacterial and fungal variation, respectively (*p* = 0.001). Treatment effects again emerged only in compartment-specific analyses, particularly in the phyllosphere, whereas rhizosphere communities were primarily structured by phenological stage.

### Compartment-specific rootstock effects on grapevine-associated bacterial communities

**Fig. 1:**
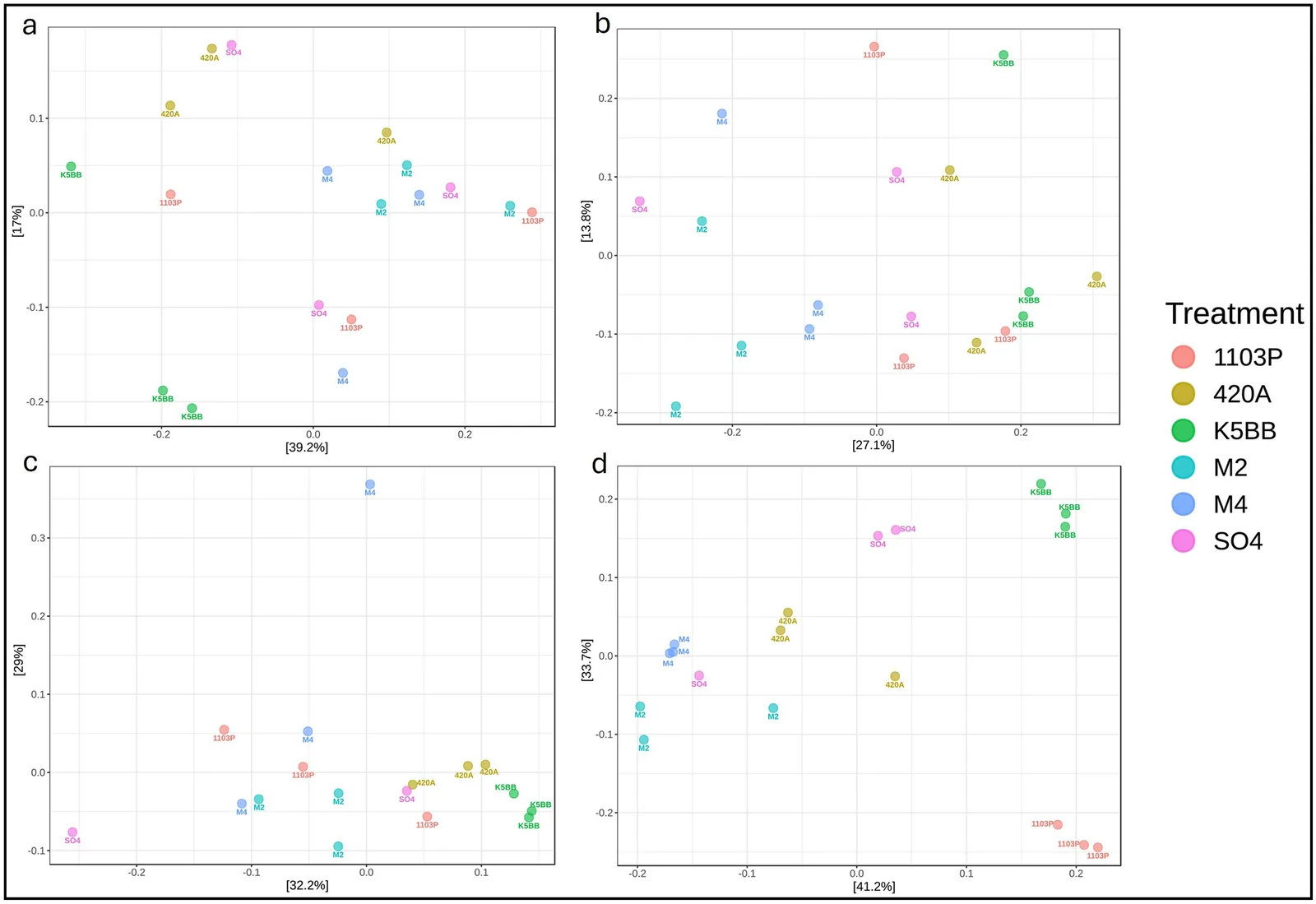
PCoA of Bray–Curtis dissimilarities showing β-diversity of phyllosphere bacterial communities across rootstock treatments at flowering (**a**, *p* = 0.049; **c**, *p* = 0.002) and veraison (**b**, *p* = 0.01; **d**, *p* = 0.001) in 2023 and 2024, respectively (*n* = 3)

In the phyllosphere, bacterial β-diversity analysis consistently revealed a significant effect of treatment across both phenological stages and years (*p* < 0.05; Fig. 1). In contrast, no significant treatment-related differences were detected in the rhizosphere of bacterial communities. Moreover, LEfSe analysis identified several rootstock-specific bacteria biomarkers (Supplementary Information 1, SI 1, Figs S1–S8).

### Season 2023

#### Phyllosphere

**Fig. 2:**
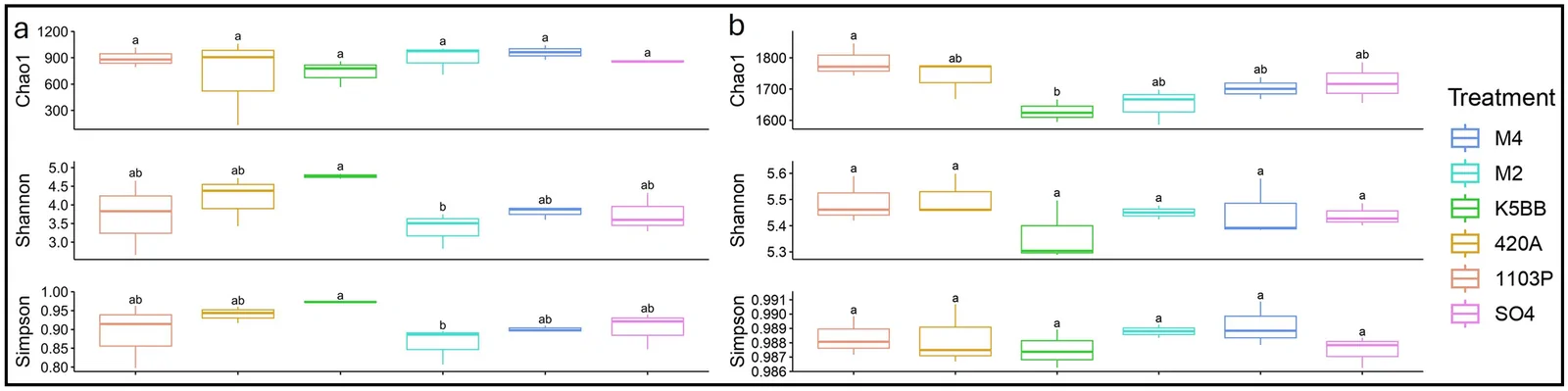
Bacteria α-diversity (Chao1, Shannon, Simpson) of phyllosphere (**a**) and rhizosphere (**b**) at flowering in 2023 (*p* < 0.05). Different letters indicate significant differences among treatments within each diversity index according to Welch’s *t*-test/ANOVA followed by pairwise post hoc comparisons (*p* < 0.05; *n* = 3)

M2 displayed reduced bacterial α-diversity compared to K5BB, as indicated by the Shannon and Simpson indices (Fig. 2a) at flowering. No other α-diversity differences were detected. Regarding community composition, bacterial β-diversity differed significantly among treatments at both flowering and veraison (PERMANOVA, *R*^2^ = 0.40, *p* = 0.049; *R*^2^ = 0.41, *p* = 0.01, respectively) (Fig. 1a and b, respectively).

PCoA ordination revealed that Axis 1 explained 39.2% and 27.1% of the variation, while Axis 2 accounted for 17.0% and 13.8% at flowering and veraison, respectively. Notably, M2 and M4 consistently formed separate clusters and were positively correlated with Axis 1 at flowering but negatively correlated with Axis 1 at veraison. At flowering, *Sphingomonas* spp. showed higher relative abundance in M2 (28%) compared to K5BB (8%), while the other rootstocks showed values around 20%. *Hymenobacter* spp. accounted for 35.4%, 27.7%, and 27.0% of the bacterial community in M2, M4, and 1103P, respectively, whereas the other rootstocks remained below 20%. *Bacillus* spp. showed higher relative abundance in K5BB, 420 A, and 1103P (7.4%, 5.5%, and 4.2%, respectively) compared to < 2% in the remaining rootstocks. At veraison, *Pantoea* spp. dominated in 420 A (34.9%), whereas *Methylobacterium* spp. showed higher relative abundance in M2 and M4 (15.2% and 12.9%, respectively).

Differential abundance analysis (LEfSe) identified distinct bacterial biomarker genera across treatments, with 13 and 16 significant features detected at flowering and veraison, respectively (SI 1, Figs. S1 and S2). At flowering, biomarkers included Vicinamibacteraceae, Chloroflexi, *Corynebacterium*, and *Microvirga* in K5BB; *Nocardioides* and Limnochordaceae in 420 A; and *Romboutsia* in SO_4_ (linear discriminant analysis, LDA = 4.6). At veraison, biomarkers included *Novosphingobium* spp. in 1103P; UTCFX1, S085, and *Desertifilum* spp. in K5BB; and Obscuribacteraceae in M4.

#### Rhizosphere

**Fig. 3:**
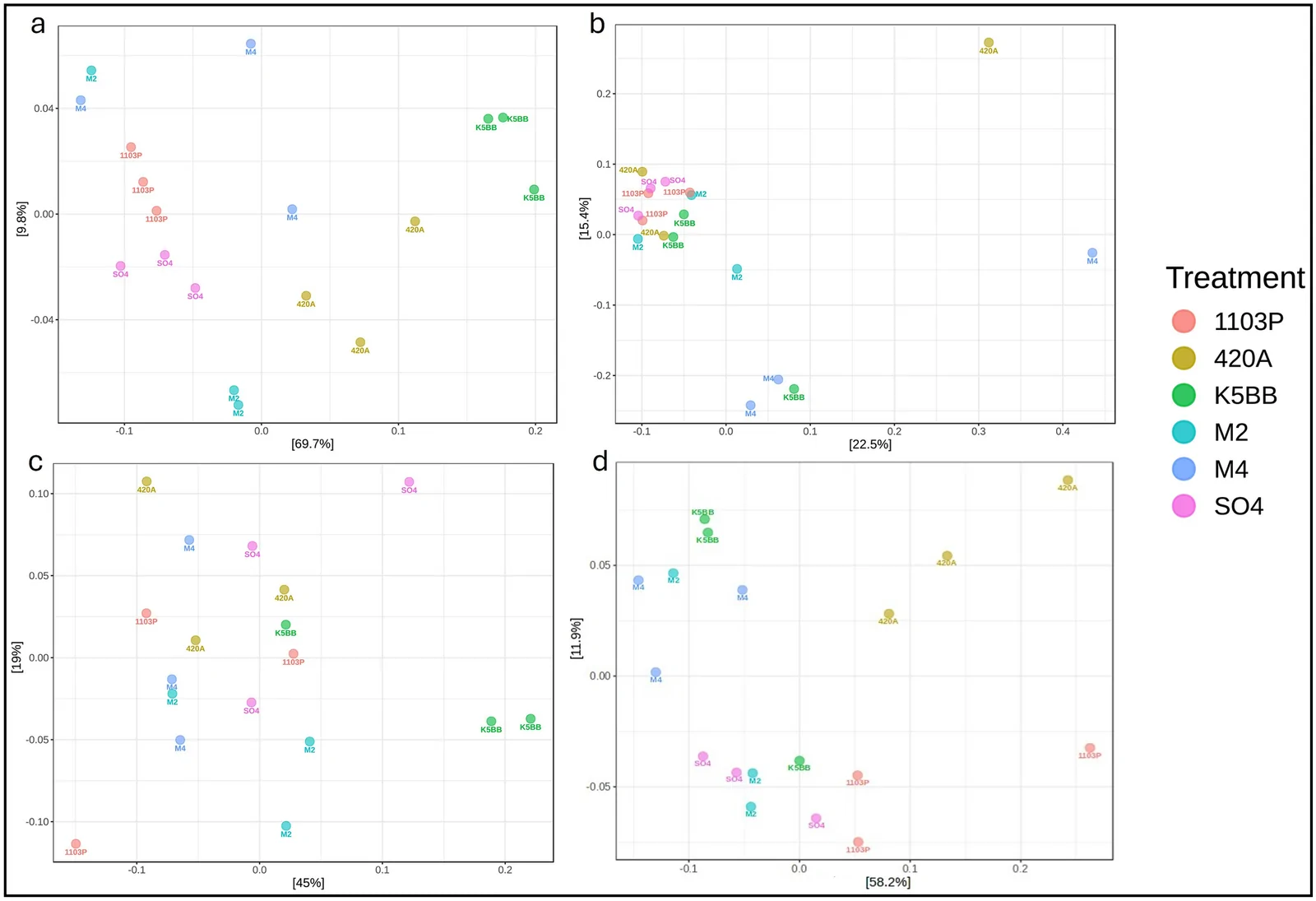
PCoA of Bray–Curtis dissimilarities showing the β-diversity of fungal communities across treatments in the phyllosphere at veraison (**a**, *p* = 0.001, *R*^2^ = 0.72) in 2023, rhizosphere at flowering in 2023 (**b**, *p* = 0.006, *R*^2^ = 0.38) and 2024 (**d**, *p* = 0.006, *R*^2^ = 0.55), and in the phyllosphere at flowering in 2024 (**c**, *p* = 0.008, *R*^2^ = 0.50) (*n* = 3)

In terms of α-diversity, no major differences were observed among treatments when considering the different indices (Chao1, Shannon, and Simpson). The only significant difference detected was a lower richness (Chao1) in K5BB compared to 1103P (Fig. 3b). LEfSe analysis identified specific bacterial biomarkers associated with individual rootstocks (SI 1, Figs. S5 and S6). Notably, *Nitrospira* spp. were associated with M2 at flowering.

### Season 2024

#### Phyllosphere

M2 and M4 displayed reduced bacterial diversity compared to 1103P and K5BB according to the Simpson index. No other α-diversity differences were detected. The bacterial community composition showed significant differences among treatments at both flowering and veraison (PERMANOVA, *R*^2^ = 0.49, *p* = 0.002; *R*^2^ = 0.82, *p* = 0.001, respectively) (Fig. 1c and d, respectively).

PCoA ordination revealed that Axis 1 explained 32.2% and 41.2% of the variation, while Axis 2 accounted for 29.0% and 33.7% at flowering and veraison, respectively. At flowering, all treatments were negatively correlated with Axis 2. At veraison, a clear separation emerged, with K5BB, SO4, 420 A, and M4 positively correlated with Axis 2, whereas M2 and 1103P were negatively correlated. At flowering, *Pseudomonas* spp. showed higher relative abundance in M4 (12.4%). At veraison, *Hymenobacter* spp. were strongly enriched in 1103P (46.9%) compared to < 15% in the other treatments. β-diversity analysis consistently confirmed a significant effect of treatment across both phenological stages and years (*p* < 0.05; Fig. 3).

LEfSe analysis identified distinct bacterial biomarker genera across treatments, with 8 and 34 significant features detected at flowering and veraison, respectively (SI 1, Figs. S3 and S4). At flowering, fewer discriminant taxa were detected, with *Massilia* (LDA = 4.5) associated with M2. At veraison, discriminant taxa included *Pantoea* spp. and *Spirosoma* spp. in M4; *Kineococcus* spp. in 420 A; *Mucilaginibacter* spp. in 1103P; *Bacillus* sp. in SO4; and several genera in K5BB, including *Methylobacterium-Methylorubrum*, *Quadrisphaera*, *Roseomonas*, *Abditibacterium*, *Marisediminicola*, *Marmoricola*, and *Nocardioides*. Notably, *Sphingomonas* sp. (LDA > 5) and *Pseudomonas* sp. were associated with M2.

#### Rhizosphere

No significant differences were detected in terms of α-diversity. LEfSe analysis identified specific bacterial biomarkers associated with individual rootstocks (SI 1, Figs. S7 and S8). Notably, *Nitrospira* spp. were associated with M4 at veraison.

### Rootstock genotype differentially shapes fungal communities across grapevine compartments

No significant differences in richness and diversity indices were detected among treatments in 2023, whereas significant treatment effects emerged in 2024. Fungal β-diversity analysis revealed a significant treatment effect, particularly at flowering (*p* < 0.05; Fig. 3). LEfSe analysis identified several rootstock-specific fungal biomarkers (SI 1, Figs. S9–S16).

### Season 2023

#### Phyllosphere

Fungal community composition in the phyllosphere was significantly affected by rootstock genotype at veraison (Fig. 3a; PERMANOVA, *R*^2^ = 0.72, *p* = 0.001). The first two PCoA axes explained 69.7% and 9.8% of the total variation, respectively.

LEfSe analysis identified *Peniophora* sp. as a biomarker associated with K5BB at flowering (SI 1, Fig. S9).

#### Rhizosphere

Fungal richness (Chao1) was lower in M4 and 1103P compared to SO4 (data not shown). The fungal community composition was significantly influenced by rootstock genotype at flowering (Fig. 3b; PERMANOVA, *R*^2^ = 0.38, *p* = 0.006), with Axis 1 and Axis 2 explaining 22.5% and 15.4% of the total variation, respectively.

LEfSe analysis revealed distinct fungal biomarkers among treatments (Suppl. Information SI, Fig. S13 and S14). At flowering, 1103P was associated with *Preussia* spp., M2 with *Minimedusa* spp. and SO4 with *Pseudoarthrographis* app (SI 1, Fig. S13). At veraison, *Waitea* spp., *Alternaria* spp., and *Sphaerobolus* spp. were enriched in 420 A; *Mortierella* spp. in M2; *Solicoccozyma* spp. and *Curvularia* spp. in M4; and *Mucor* spp. and *Polyschema* spp. in SO4 (SI 1, Fig. S14).

### Season 2024

#### Phyllosphere

**Fig. 4:**
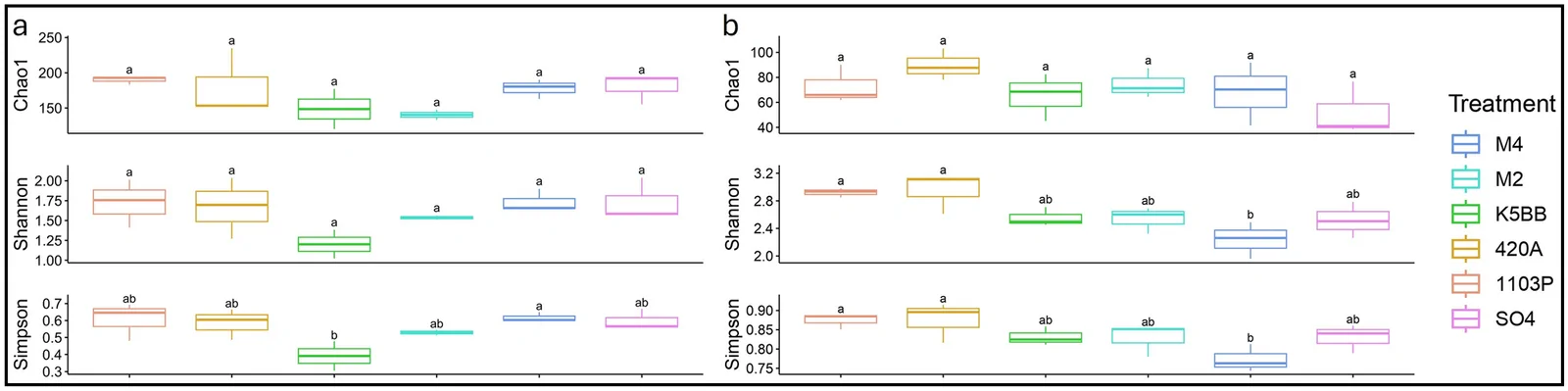
Fungal α-diversity (Chao1, Shannon, Simpson) of phyllosphere (**a**) and rhizosphere (**b**) at flowering in 2024 (*p* < 0.05; *n* = 3)

Simpson diversity showed lower values in K5BB than in M2 (Fig. 4a). Rootstock genotype significantly shaped phyllosphere fungal communities at veraison (Fig. 3c; PERMANOVA, *R*^2^ = 0.50, *p* = 0.008). The first two PCoA axes explained 45% and 19% of the total variation, respectively. At veraison*, Aureobasidium* spp. was associated with 1103P, whereas *Articulospora* sp. was enriched in M2 (SI 1, Fig. S12).

#### Rhizosphere

Both Shannon and Simpson indices showed lower α-diversity in M2 compared to 420 A and 1103P (Fig. 4b). Rhizosphere fungal community composition was significantly influenced by rootstock genotype at flowering (Fig. 3d; PERMANOVA, *R*^2^ = 0.55, *p* = 0.006). Axis 1 and Axis 2 explained 58.2% and 11.9% of the total variation, respectively. At flowering, 1103P was associated with *Cephalotrichum* spp. and *Pseudoarthrographis* sp.; the latter also identified as a biomarker for SO4 (SI 1, Fig. S15). Additionally, *Knufia* spp. was identified as a biomarker in M2 (SI 1, Fig. S16) at veraison.

### PICRUSt and FUNGuild-based functional prediction

PICRUSt was applied to predict bacterial functions across phenological stages and plant compartments. In the rhizosphere, no significant differences were observed, whereas in the phyllosphere at veraison the PCA revealed clear clustering among treatments (SI 1, Fig. S17). PC1 accounted for 55.6% and 62.4% of the variance in 2023 and 2024, respectively, and pathways were significantly modulated in both years.

The major effect was observed in 2024, with 270 significantly modulated pathways across the treatments (SI 2, Table S1). Pairwise comparisons revealed significant differences (*p* < 0.05) among treatments. SO4 showed no significant differences with M2, 420 A, or K5BB, but 30 and 12 pathways with 1103P and M4, respectively. M4 displayed the largest shifts, with 291, 233, and 119 pathways significantly modulated when compared with 1103P, K5BB, and 420A. M2 showed intermediate divergence (102 with 1103P, 113 with K5BB, 29 with M4, and 8 with 420 A). 1103P differed from 420 A and K5BB with 147 and 178 pathways, while 420 A and K5BB differed by 106. Overall, M4 and 1103P drove the strongest functional shifts, whereas SO4, M2, 420 A, and K5BB showed lower divergence.

Several pathways showed higher relative abundance in 1103P, particularly those related to cell-envelope and membrane biosynthesis (e.g., lipid A, peptidoglycan and O-antigen pathways) and core carbon and NAD metabolism. Pathways involved in the degradation of aromatic and phenolic compounds (such as glucarate and galactarate degradation) also varied across treatments, with 1103P and M4 showing a distinct, though moderate, enrichment.

FUNGuild was used to analyze the nutritional and functional groups of fungal communities across treatments. Both single and multi-affiliated assignments were detected, with trophic modes including pathotroph, saprotroph, and symbiotroph, as well as guild combinations such as plant pathogen-wood saprotroph, plant saprotroph-undefined saprotroph, and endophyte-plant saprotroph- undefined saprotroph. In 2024, no differences among treatments were detected in the rhizosphere, whereas in the phyllosphere, the mixed trophic mode Pathotroph–Saprotroph–Symbiotroph showed a significant overall difference among treatments (Kruskal–Wallis, *p* = 0.019). However, Dunn’s post hoc pairwise comparisons revealed no significant pairwise differences after Benjamini–Hochberg correction.

### Multivariate associations between microbial community structure, physiological traits and vegetative growth

In 2023, at veraison, leaf area tended to be associated with phyllosphere bacterial community structure (envfit, *R*^2^ = 0.29, *p* = 0.07), whereas node number showed a marginally significant association (*R*^2^ = 0.32, *p* = 0.05) (SI 2, Table S2A). Rhizosphere bacterial community structure was not significantly influenced by rootstock treatment but instead followed a physiological gradient, with Ψ_stem_ explaining a substantial proportion of the variation (envfit, *R*^2^ = 0.49, *p* < 0.01). In the same season and phenological stage, rhizosphere fungal communities were mainly associated with E and g_s_, which explained most of the variation (envfit, *R*^2^ = 0.50, *p* < 0.01 and *R*^2^ = 0.40, *p* < 0.05, respectively) (SI 2, Table S2B).

In 2024, at veraison, envfit analysis revealed significant associations between phyllosphere bacterial community structure and the physiological parameters A, WUEe, and Ψ_stem_, as well as the vegetative growth traits leaf area (LA), number of nodes, and pruning weight (*p* < 0.05; *R*^2^ > 0.40) (SI 2, Table S2A). Mantel test showed positive, but non-significant trends, correlation between physiological and bacterial distance matrices for Ψ_stem_ (*r*^2^ = 0.24, *p* = 0.060), LA (*r*^2^ = 0.23, *p* = 0.060), Node count (*r*^2^ = 0.24, *p* = 0.060) and pruning weight (*r*^2^ = 0.21, *p* = 0.060). No significant correlations were found for fungal communities.

## Discussion

The comparison of different rootstock genotypes, including the recently developed M2 and M4 and traditional commercial rootstocks, under contrasting seasonal conditions provided a useful framework to assess whether rootstock-related host variation extends beyond plant performance to shape grapevine-associated microbial communities. Although year, plant compartment, and phenological stage represented the primary drivers of microbiome variation, our data indicate that, within comparable environmental contexts, rootstock genotype exerted a distinct and measurable effect on grapevine-associated microbial communities. The effect was stronger for phyllosphere bacteria, whose communities differed significantly between treatments in both years and phenological stages, whereas fungal assemblages showed a more variable response across compartments and seasons. This pattern may reflect the higher sensitivity of the phyllosphere to canopy-associated microenvironmental conditions, potentially influenced by rootstock-induced differences in vine vigor and physiological activity (Warschefsky et al. 2016).

Seasonal variation in rainfall and heat accumulation markedly influenced vine physiology between treatments and, consequently, the assembly of microbial communities. At flowering, Ψ_stem_ averaged around − 0.6 MPa, indicating that vine water status was not limiting (Choné 2001). By veraison, contrasting seasonal patterns became evident. In 2023, 420 A, K5BB, 1103P, and SO4 experienced severe drought stress, while M2 and M4 maintained a moderate drought stress. Despite these differences, all vines showed a marked reduction in leaf gas-exchange activity. Although rhizosphere bacterial β-diversity did not differ significantly among treatments this year, variation in Ψ_stem_ likely explained part of the observed community patterns. In contrast, rhizosphere fungal communities showed significant treatment-related shifts at veraison, suggesting that fungi were more sensitive than bacteria to differences in vine water status.

The higher rainfall recorded in 2024 likely resulted in a milder water deficit, allowing the drought-tolerant rootstocks M2, M4, and 1103P to better express their physiological advantages, consistent with the higher photosynthetic performance previously reported for these genotypes under moderate water stress (Bianchi et al. 2018). These physiological differences among rootstocks likely contributed to the distinct clustering observed in the phyllosphere bacterial communities. Accordingly, the significant correlations of bacterial communities with A, WUEe and Ψ_stem_ at veraison in 2024 further indicate that variation in the phyllosphere microbiome is closely linked to vine physiological status, consistent with the host-dependent dynamics described by Vorholt (2012).

Across years and phenological stages, microbial richness showed treatment-dependent fluctuations mainly in the rhizosphere, particularly at flowering, likely reflecting enhanced root activity and the increased release of root-derived organic compounds during this period of high metabolic demand (Kulmann et al. 2022). Rootstock effects on microbial α-diversity were generally modest and varied according to the plant compartment and sampling time, while our observations are consistent with the limited influence on rhizosphere diversity reported by Zuzolo et al. (2023). In contrast, M2 and M4 tended to harbor less even bacterial communities in the phyllosphere, suggesting that these rootstocks may exert a stronger selective effect on aboveground microbiota. Differences in microbial richness and community composition were detected at flowering, despite similar vine water status across treatments in both years. This suggests that intrinsic rootstock traits, such as metabolic activity, root exudation patterns, or differences in nutrient uptake, may have played a key role in shaping the associated microbiome (Marasco et al. 2018; Berlanas et al. 2019; Dries et al. 2021). These findings suggest that, beyond environmental and water-related factors, rootstock-specific physiological mechanisms contribute to the recruitment and modulation of microbial communities, particularly during early stages of vine development when root metabolic activity is most intense (Chaparro et al. 2014; Zarraonaindia et al. 2015).

While several studies have shown that rootstocks can shape rhizosphere bacterial diversity (D’Amico et al. 2018; Marasco et al. 2018; Zuzolo et al. 2023), in our study this effect was not evident in the rhizosphere but was instead more pronounced in the phyllosphere. Considering that the same scion was used across all treatments, these differences are likely attributable to the rootstock genotype rather than scion-related effects. A plausible explanation is that rootstocks modulated vine vigor and canopy architecture, reflected by differences in vegetative growth across treatments in both 2023 and 2024, potentially leading to distinct leaf-associated microenvironmental conditions that may have influenced phyllosphere assembly at veraison. Such effects may also be context-dependent. For instance, Berlanas et al. (2019) observed no variation in rhizosphere microbiomes among rootstock genotypes in a 7-year-old vineyard, comparable in age to ours, whereas significant effects were detected in a 25-year-old vineyard. This may explain the limited rootstock influence on rhizosphere bacterial communities in our case. The rhizosphere likely represents a more stable niche, primarily structured by soil properties, whereas the phyllosphere is more dynamic and responsive to plant physiological status and microclimatic conditions (Vorholt 2012), reflecting rootstock-driven differences in water and nutrient fluxes and scion vigor.

In 2023, the drier season, M2 and M4 showed higher relative abundance of *Methylobacterium* spp. in the phyllosphere, which coincided with their better water status. Because *Methylobacterium* thrives under active leaf physiology and contributes to plant stress tolerance through mechanisms such as phytohormone production and nutrient acquisition (Zhang et al. 2021), its enrichment likely reflects, and possibly reinforces, the comparatively lower stress experienced by these treatments. Interestingly, *Sphingomonas* sp., identified as a biomarker of M2 in the phyllosphere, includes taxa known to promote plant growth through the production of phytohormones such as indole−3-acetic acid (IAA) and gibberellins (Khan et al. 2014) and to exert biocontrol activity (Innerebner et al. 2011). Although its ecological role on grapevine leaves remains to be clarified, its enrichment in M2 suggests a potential contribution to host-microbe interactions at the leaf surface. Similarly, the occurrence of *Aureobasidium* spp. as a biomarker in 1103P points to potential biocontrol functions within the phyllosphere, while the enrichment of *Mortierella* spp. in M2 supports enhanced belowground nutrient cycling (Osorio and Habte 2001).

Although amplicon sequencing cannot directly resolve microbial functions, predictive tools such as PICRUSt2 and FUNGuild can infer functional guilds and metabolic potentials, providing insights into community functioning despite their limitations. Unlike Labarga et al. (2025), we did not detect any significant differences in predicted rhizosphere bacterial functionality among rootstocks, supporting previous study reporting a limited influence of rootstock genotype on microbial functional profiles (Marasco et al. 2018). In contrast, the phyllosphere exhibited a stronger functional response to rootstock genotype, likely reflecting the greater taxonomic differences observed in this compartment. FUNGuild, however, proved less informative, as a substantial proportion of sequences remained unassigned, consistent with Lailheugue et al. (2024). According to PICRUSt2, phyllosphere samples clustered into three groups (M2-M4, 420A-SO4-K5BB, and 1103P), reflecting differences in genetic background, associated physiological traits, and leaf area. Enriched pathways among these groups suggested moderate functional differentiation, including aromatic compound metabolism in 1103P and M4. While these predicted functions cannot be directly linked to fruit composition within the scope of this study, they may reflect microbial functional potentials relevant to the turnover of plant-derived compounds in the canopy environment (Cueva et al. 2012). Further studies are needed to determine whether and how phyllosphere-associated microbial communities relate to microbial assemblages on grape berries and whether genotype-associated microbial patterns may influence early processing stages in ways that are ecologically or enologically relevant (Barata et al. 2012; Griggs et al. 2025). This is particularly relevant given the growing recognition of the grapevine microbiome as a biological component of terroir.

## Conclusion

Distinct rootstock genotypes were consistently associated with shifts in grapevine-associated microbial communities, particularly in the phyllosphere. Although year and seasonal environmental conditions explained most of the observed variation, rootstock genotype exerted a measurable effect within comparable soil, scion, and management contexts. At the same time, differences in vine water status, photosynthetic performance, and canopy development among treatments indicate that rootstock genotype also modulated key plant functional traits, which may in turn have contributed to microbial assembly through changes in plant functioning and associated microclimatic conditions.

The recurrent association of specific microbial groups with individual treatments further indicates that rootstock genotype may act as a selective filter, structuring microbial assemblages both above- and belowground. These patterns highlight the role of rootstock-related processes, including water regulation, nutrient acquisition, and canopy architecture, in shaping the grapevine microbiome beyond the rhizosphere compartment. A key challenge in disentangling these interactions lies in the inherent structural complexity and spatial heterogeneity of vine root systems, which complicates fine-scale in situ characterization. Addressing this complexity will require integrative frameworks that combine genome-resolved and activity-based approaches to link community shifts with functional outcomes and to identify robust beneficial taxa. Such analyses should adopt a whole-plant perspective, encompassing both below- and aboveground compartments. Expanding this integrative view will deepen our understanding of rootstock-associated microbial recruitment, its physiological drivers, and its ecological and potentially enological relevance for resilient and sustainable viticulture, reinforcing the strategic importance of rootstock choice under ongoing climate change.

## Supplementary Information

Below is the link to the electronic supplementary material.

Supplementary Information 1 (DOCX 2282 kb)

Supplementary Information 2 (XLSX 221 kb)

## Data Availability

Sequence data were submitted to the National Center for Biotechnology Information Sequence Read Archive (BioProject ID PRJNA1372066).
