# Supplementary material for: Rootstock genotype shapes vine functional traits and associated microbiomes, with predominant shifts in the phyllosphere
Source: Planta. 2026 Sep 8;264(4):122. doi: 10.1007/s00425-026-05132-6 (PMC13553729; doi:10.1007/s00425-026-05132-6)
Supplement: Supplementary file 1 [file 425_2026_5132_MOESM1_ESM.docx]

**Rootstock genotype shapes vine functional traits and associated microbiomes, with predominant shifts in the phyllosphere**

Ginevra Canavera^1,2,3^, Filippo Vaccari^1^, Matteo Gatti^2^, Edoardo Puglisi^1,3^*, Tommaso Frioni^2,3^

^1^Department for Sustainable Food Process, Università Cattolica del Sacro Cuore, Via Emilia Parmense 84, 29122 Piacenza, Italy

^2^Department of Sustainable Crop Production, Università Cattolica del Sacro Cuore, Via Emilia Parmense 84, 29122 Piacenza, Italy

^3^National Research Centre for Agricultural Technologies (Agritech Foundation, PNRR M4C2), Naples, Italy

*Corresponding: [edoardo.puglisi@unicatt.it](mailto:edoardo.puglisi@unicatt.it)

**Supplementary Information 1**

LEFSE analysis of phyllosphere and rhizosphere of bacterial and fungal communities in 2023 and 2024 and PCA of predicted bacterial functional pathways in the phyllosphere at veraison in 2023 and 2024

**Fig. S1** LEfSe analysis of phyllosphere bacterial communities at flowering in 2023

Treatment

Treatment

**Fig. S2** LEfSe analysis of phyllosphere bacterial communities at veraison in 2023

**Fig. S3** LEfSe analysis of phyllosphere bacterial communities at flowering in 2024

Treatment

Treatment

**Fig. S4** LEfSe analysis of phyllosphere bacterial communities at veraison in 2024

**Fig. S5** LEfSe analysis of rhizosphere bacterial communities at flowering in 2023

Treatment

**Fig. S6** LEfSe analysis of rhizosphere bacterial communities at veraison in 2023

Treatment

**Fig. S7** LEfSe analysis of rhizosphere fungal communities at flowering in 2024

Treatment

**Fig. S8** LEfSe analysis of rhizosphere fungal communities at veraison in 2024

Treatment

Treatment

**Fig. S9** LEfSe analysis of phyllosphere fungal communities at flowering in 2023

Treatment

Treatment

**Fig. S10** LEfSe analysis of phyllosphere fungal communities at veraison in 2023

**Fig. S11** LEfSe analysis of phyllosphere fungal communities at flowering in 2023

Treatment

Treatment

**Fig. S12** LEfSe analysis of phyllosphere fungal communities at veraison in 2023

**Fig. S13** LEfSe analysis of rhizosphere fungal communities at flowering in 2023

Treatment

Treatment

**Fig. S14** LEfSe analysis of rhizosphere fungal communities at flowering in 2023

**Fig. S15** LEfSe analysis of rhizosphere fungal communities at flowering in 2024

Treatment

Treatment

**Fig. S16** LEfSe analysis of rhizosphere fungal communities at veraison in 2024

**Fig. S17** Principal Component Analysis (PCA) of PICRUSt2-predicted bacterial functional pathways in the phyllosphere at veraison in 2023 (a) and 2024 (b).

Treatment
